# Towards risk stratification and prediction of disease severity and mortality in COVID-19: Next generation metabolomics for the measurement of host response to COVID-19 infection

**DOI:** 10.1371/journal.pone.0259909

**Published:** 2021-12-01

**Authors:** Paulo D’Amora, Ismael Dale C. G. Silva, Maria Auxiliadora Budib, Ricardo Ayache, Rafaela Moraes Siufi Silva, Fabricio Colacino Silva, Robson Mateus Appel, Saturnino Sarat Júnior, Henrique Budib Dorsa Pontes, Ana Carolina Alvarenga, Emilli Carvalho Arima, Wellington Galhano Martins, Nakal Laurenço F. Silva, Ricardo Sobhie Diaz, Marcia B. Salzgeber, Anton M. Palma, Steven S. Evans, Robert A. Nagourney

**Affiliations:** 1 Department of Gynecology, Molecular Gynecology and Metabolomics Lab, College of Medicine of the Federal University of São Paulo (EPM-UNIFESP), São Paulo, São Paulo, Brazil; 2 Nagourney Institute, Long Beach, California, United States of America; 3 Metabolomycs, Inc., Long Beach, California, United States of America; 4 Nagourney Cancer Institute, Long Beach, California, United States of America; 5 Department of Emergency and Intensive Care Unit, CASSEMS Hospital, Campo Grande, Mato Grosso do Sul, Brazil; 6 Department of Medicine, Infectious Disease Division, Retrovirology Laboratory, College of Medicine of the Federal University of São Paulo (EPM-UNIFESP), São Paulo, São Paulo, Brazil; 7 Institute for Clinical and Translational Science (ICTS), University of California Irvine (UCI), Irvine, California, United States of America; 8 Todd Cancer Institute, Memorial Medical Center of Long Beach, Long Beach, California, United States of America; 9 Department of Obstetrics and Gynecology, University of California Irvine (UCI), Orange, California, United States of America; National Research Council of Italy, ITALY

## Abstract

This study investigated the association between COVID-19 infection and host metabolic signatures as prognostic markers for disease severity and mortality. We enrolled 82 patients with RT-PCR confirmed COVID-19 infection who were classified as mild, moderate, or severe/critical based upon their WHO clinical severity score and compared their results with 31 healthy volunteers. Data on demographics, comorbidities and clinical/laboratory characteristics were obtained from medical records. Peripheral blood samples were collected at the time of clinical evaluation or admission and tested by quantitative mass spectrometry to characterize metabolic profiles using selected metabolites. The findings in COVID-19 (+) patients reveal changes in the concentrations of glutamate, valeryl-carnitine, and the ratios of Kynurenine/Tryptophan (Kyn/Trp) to Citrulline/Ornithine (Cit/Orn). The observed changes may serve as predictors of disease severity with a (Kyn/Trp)/(Cit/Orn) Receiver Operator Curve (ROC) AUC = 0.95. Additional metabolite measures further characterized those likely to develop severe complications of their disease, suggesting that underlying immune signatures (Kyn/Trp), glutaminolysis (Glutamate), urea cycle abnormalities (Cit/Orn) and alterations in organic acid metabolism (C5) can be applied to identify individuals at the highest risk of morbidity and mortality from COVID-19 infection. We conclude that host metabolic factors, measured by plasma based biochemical signatures, could prove to be important determinants of Covid-19 severity with implications for prognosis, risk stratification and clinical management.

## Introduction

On December 31, 2019, a cluster of atypical pneumonia cases was reported in Wuhan, Hubei province, China. By mid-January 2020, the first case of this SARS/MERS variant dubbed COVID-19 was reported in the United States. Over time, this coronavirus variant rapidly spread around the world resulting in one of worst pandemics in modern history [1]. While 80% of infected individuals show mild symptoms, approximately 20% progress to pneumonia, ARDS, multi-organ failure or death [2], with the highest risk of symptoms and complications occurring among persons with pre-existing co-morbidities including obesity, diabetes mellitus, hypertension, and cardiovascular disease [3]. The association between these cardio-metabolic conditions and disease severity suggested the possibility of a metabolic predisposition [4].

We had previously examined the association between retroviral infection with the HIV-Lentivirus and the levels of 186 different metabolites quantified using tandem mass spectrometry (MS/MS) conducted upon plasma. We identified metabolomic signatures that could distinguish HIV rapid-progressors and immunologic-non-responders from controls, suggesting that host metabolic factors strongly influenced the severity of HIV infection [5].

To determine whether similar metabolic signatures are found in patients with COVID-19 infection and to examine the impact of these signatures upon clinical outcome, we conducted a prospective study on the plasma of 82 patients positive for COVID-19 infection by RT-PCR and compared the results with 31 plasma samples from healthy volunteers using quantitative tandem MS/MS.

## Materials and methods

### Study design and patient accrual

A cross sectional and prospective observational study was conducted at CASSEMS General Hospital in the city of Campo Grande, Mato Grosso do Sul State (southwestern Brazil), in collaboration with investigators from the Federal University of São Paulo (EPM-UNIFESP), São Paulo, Brazil; Nagourney Institute and Metabolomycs, Inc., both in Long Beach, California, USA.

The protocol was approved by the Institutional Review Board from the Federal University of São Paulo (CEP/UNIFESP—approval CAAE: 37348020.3.0000.5505) and was conducted in compliance with the World Medical Association Declaration of Helsinki. Written informed consents were obtained from all participants.

All patients who were accrued to the study tested positive for SARS-CoV2 and were followed for clinical outcome, categorized as mild (n = 20), moderate (n = 32) or severe (n = 30) according to World Health Organization classification of severity [6]. The control group (n = 31) was composed of healthy volunteers who tested negative for SARS-CoV2. All patients and controls submitted an EDTA-purple-top tube collected from peripheral blood samples, obtained at the time of protocol accrual. All patients and control subjects provided written informed consent for participation in the study protocol.

### Inclusion and exclusion criteria

Between November 30^th^, 2020, and January 20^th^, 2021, all patients over the age 18 who presented to the Cassems Hospital for evaluation of respiratory symptoms, who tested positive for COVID-19 by RT-PCR were eligible for inclusion.in the study. Patients positive for COVID-19 were stratified as mild, moderate, and severe/critical according to WHO criteria [6]. Healthy volunteers (consisting of CASSEMS Hospital healthcare providers) who tested negative for SARS-CoV2 served as controls.

### Clinical and laboratory data assessment

Nasal and pharyngeal swab specimens were collected either in the emergency room (ER) or during hospitalization, and a confirmed case of COVID-19 was defined as having detectable SARS-CoV-2 virus on real-time reverse-transcriptase polymerase chain reaction (RT-PCR) assay, carried out according to validated protocols [7].

Clinical data was extracted by chart review from physician notes and medical records in the CASSEMS healthcare database. Data on symptoms and vital signs were collected at initial presentation in the ER or as part of the admission history. Data on past medical history and comorbidities were collected from medical records. Fever was defined as forehead temperature >37.4°C (>99.3 F), and hypoxemia was defined as pulse oximetry reading from finger oximeter <90%. Hypotension was defined as mean arterial pressure (MAP) <65 mmHg and tachycardia were defined as heart rate (HR) >100 beats per minute (bpm). All laboratory values on the day of admission or during hospitalization were collected from the Medical Records. Laboratory values included complete blood counts, blood chemistry including renal function, C-reactive protein (C-RP), d-dimer, arterial blood gas. Details of radiologic examinations such as computed tomography (CT) scanning of the chest were also collected [8, 9]. Clinical and laboratory data was collected and analyzed by the healthcare team at CASSEMS Hospital who vouched for accuracy and completeness of data and for adherence of study to protocol.

### Study outcomes

The primary composite endpoint is recovery or WHO-classified severity of illness defined as the need for mechanical ventilation, use of inotrope support, intensive care unit (ICU) admission, or death. Secondary endpoints are development of acute respiratory distress syndrome (ARDS), secondary pneumonia; acute renal failure, acute cardiac injury, and length of hospital stay [10].

### Collection of blood samples

Peripheral venous blood samples from each patient/volunteer were collected using tubes with anti-clotting factor (EDTA). Immediately after blood collection, samples were centrifuged (5 min at 4000 rpm). After centrifugation, the plasma was aliquoted, frozen, and stored at −80°C for targeted mass spectrometry analysis.

### Metabolomic analysis workflow

**Fig 1:**
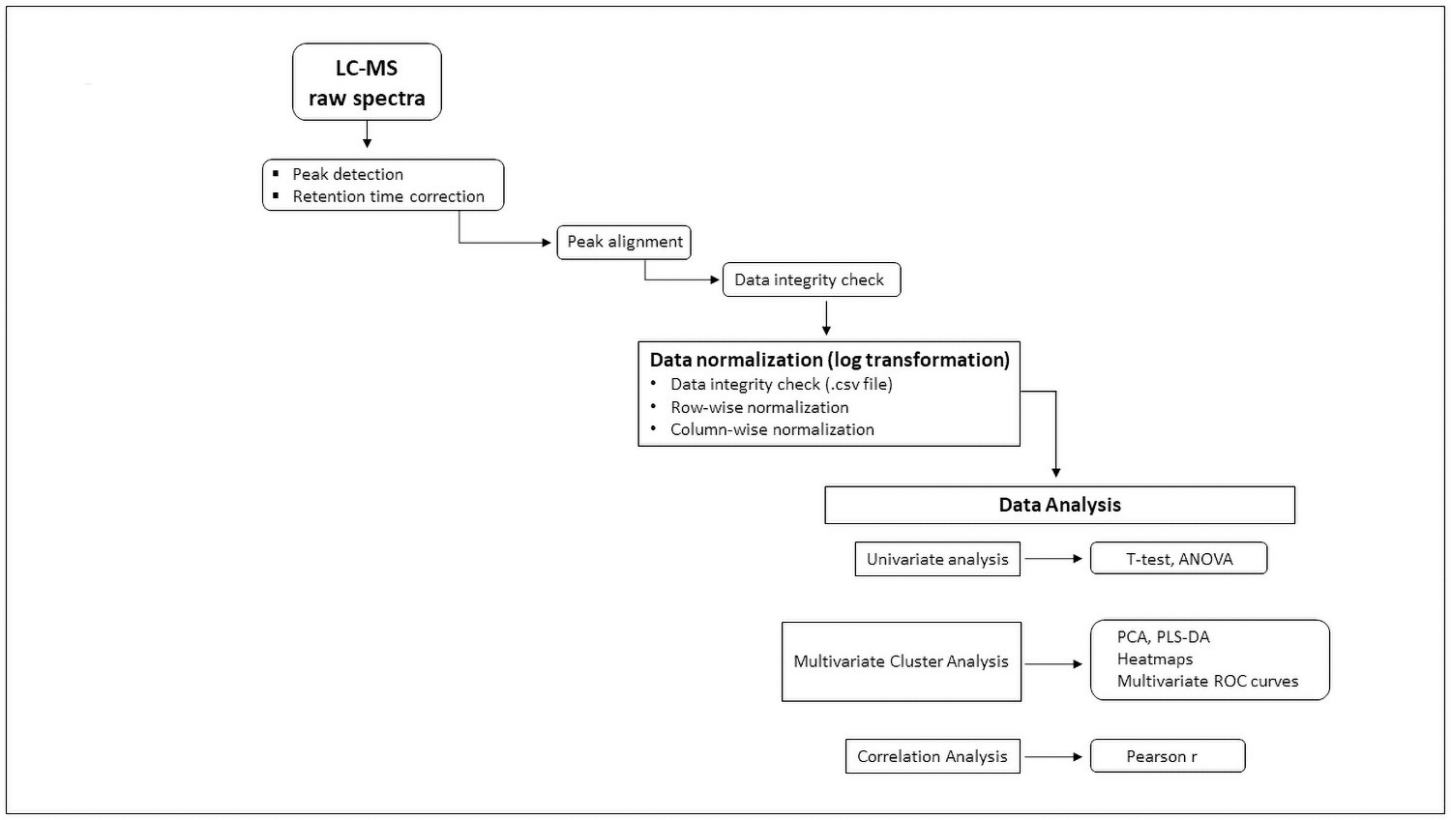
Flowchart illustrating workflow and data processing. Individual metabolite absolute concentrations measured by targeted mass spectrometry (MS/MS) transmitted in.csv data-files were log transformed for normalization and then uploaded into MetaboAnalyst 5.0 bio-informatic data analytic platform. Univariate (t-test, ANOVA), multivariate (PCA, PLS-DA, Heatmaps, Multivariate ROC Curve Analysis) and correlation coefficients (Pearson r) then applied to identify metabolites and ratios associated with COVID-19.

Fig 1 provides sample processing flowsheet from plasma receipt to liquid chromatography and mass spectrometry through data analysis.

### Targeted quantitative MS/MS analysis

In this study, targeted metabolomic analyses of plasma samples were performed using Absolute IDQ^®^ P180 kit from Biocrates (Biocrates, Life Science AG, Innsbruck, Austria). This validated targeted assay allows simultaneous detection and absolute quantification of metabolites in plasma in a high throughput manner. This kit can be used on a variety of LC-MS/MS instruments and has already been applied to many studies of human serum and plasma, including several large-scale prospective cohort studies [11–15]. Absolute quantification (μmol/L) of blood metabolites was achieved by targeted quantitative profiling of 186 annotated metabolites by electrospray ionization (ESI) tandem mass spectrometry (MS/MS) in 113 biological samples, blinded to any phenotype information, on a centralized, independent, fee-for-service basis at the quantitative metabolomics platform from BIOCRATES Life Sciences AG, Innsbruck, Austria. Briefly, a targeted profiling scheme was used to quantitatively screen for fully annotated metabolites using multiple reaction monitoring, neutral loss, and precursor ion scans. Quantification of metabolite concentrations and quality control assessment were performed with the MetIQ software package (BIOCRATES Life Sciences AG, Innsbruck, Austria), which implies proof of reproducibility within a given error range. An MS Excel file (.xls) was then generated, which contained sample identification and 186 metabolite names and concentrations with the unit of μmol/L of plasma [16].

### Metabolite panel

In total, 186 annotated metabolites were quantified using the Biocrates AbsoluteIDQ^®^ p180 kit (Biocrates Life Sciences AG, Innsbruck, Austria), being 40 acylcarnitines (ACs), 21 amino acids (AAs), 19 biogenic amines (BA), sum of hexoses (Hex), 76 phosphatidylcholines (PCs), 14 lysophosphatidylcholines (LPCs) and 15 sphingomyelins (SMs), glycerophospholipids were further differentiated with respect to the presence of ester (a) and ether (e) bonds in the glycerol moiety, where two letters denote that two glycerol positions are bound to a fatty acid residue (aa = diacyl, ae = acyl-alkyl), while a single letter indicates the presence of a single fatty acid residue (a = acyl or e = alkyl) [17].

### Validation tests

For Metabolomic Data Analysis, log-transformation was applied to all quantified metabolites to normalize the concentration distributions and uploaded into the web based analytical pipelines MetaboAnalyst 5.0 (www.metaboanalyst.ca/faces/upload/RocUploadView.xhtml) and Receiver Operating Characteristic Curve Explorer & Tester (ROCCET) available at (https://www.metaboanalyst.ca/resources/data/metabolomics2012_xia.pdf) for the generation of uni- and multivariate Receiver Operating Characteristic (ROC) curves obtained through Support Vector Machine (SVM), Partial Least Squares-Discriminant Analysis (PLS-DA) and Random Forests as well as Logistic Regression Models were used to calculate Odds Ratios of specific metabolites. ROC curves were generated by Monte-Carlo Cross Validation (MCCV) using balanced sub-sampling where two thirds (2/3) of the samples were used to evaluate the feature importance. Significant features were then used to build classification models, which were validated on the 1/3 of the samples that were left out on the first analysis. The same procedure was repeated 10–100 times to calculate the performance and confidence interval of each model. To further validate the statistical significance of each model, ROC calculations included bootstrap 95% confidence intervals for the desired model specificity as well as accuracy after 1000 permutations and false discovery rates (FDR) calculation [18].

### Statistical analysis

Sample characteristics were evaluated with continuous variables expressed as means and standard deviation and categorical variables as frequencies and percentages. Logistic regression models were fit to compare the effects of each metabolite measure as a potential predictor on each clinical outcome, both with and without control for age, sex, and BMI to estimate unadjusted and adjusted odds ratios (OR) and 95% confidence intervals (CIs) for the association between each metabolite and each outcome. P-Statistical significance was set at values P <0.05 Statistical analyses for these clinical outcome models were performed using R version 4.0.1.

## Results and discussion

The COVID-19 pandemic has had a profound impact upon every aspect of human existence with over 224,588,128 cases and 4,628,882 deaths reported by the WHO as of September 2021. The resulting disruptions have devastated economies, overwhelmed health care delivery and severely restricted international trade and travel.

The medical community’s response to COVID-19 has largely focused upon the infecting agent’s virulence, mode of transmission, infectivity, and molecular features. While we have come to understand the virus’s capacity to gain entry to the cell via the ACE-2 receptor, characterized the structure of the Spike Protein, identified mutational variants, and developed vaccines to prevent infection and transmission [19, 20], less is known about the effectiveness of the host’s response to the infection.

Severe complications of COVID-19 including coagulopathies, ARDS, hepatic and renal failure and multisystem damage are shared by other infectious processes [21].

To better understand the physiologic response of the host to COVID-19 infection we used plasma metabolic signatures to examine the intrinsic features of each patient’s mechanisms of defense. Our question being: Is it the pathogenicity of the infecting organism or the host’s response and defenses that determine the ultimate morbidity and mortality of the disease? With insights from our prior work in HIV [5] and that of Davanzo et al. [22], we explored metabolic signatures in the plasma of COVID-19 patients.

The study sample had mean age 48.6 years (SD = 12.5 years), 51% male, 75% overweight or obese and had high prevalence of comorbid health conditions, notably hypertension (33%) and diabetes mellitus (12%). COVID-related symptoms were very common at the time of presentation to the hospital, with half of the sample presenting with cough, and several other known symptoms commonly reported (fever: 43%, asthenia: 31%, dyspnea: 29% and myalgia: 29%). While the majority of patients recovered or were discharged (68%), several patients required supplemental oxygen (16%) or intubation with mechanical ventilation (16%). Sample characteristics overall and by disease severity are shown in Table 1.

**Table 1:** Patient demographics and clinical characteristics.

Variables	All Groups	Severe/Critical	Moderate	Mild	Control
	N (%)	N (%)	N (%)	N (%)	N (%)
**Total**	113 (100%)	30 (27%)	32 (28%)	20 (18%)	31 (100%)
**Demographics**					
Age (years), mean (SD)	48.58 (12.53)	56.77 (10.2)	53.25 (10.4)	43.25 (10.5)	39.29 (10.28)
Male	58 (51.3%)	23 (76.7%)	18 (56.2%)	7 (35.0%)	10 (32.3%)
Female	55 (48.7%)	7 (23.3%)	14 (43.8%)	13 (65.0%)	21 (67.7%)
**BMI categories**					
Normal	28 (24.8%)	3 (10%)	3 (9.4%)	9 (45%)	13 (41.9%)
Overweight	43 (38.1%)	12 (40%)	12 (37.5%)	6 (30%)	13 (41.9%)
Obese	42 (37.2%)	15 (50%)	17 (53.1%)	5 (25%)	5 (16.1%)
**Comorbidities**					
Cardiovascular disease	9 (8%)	7 (23.3%)	2 (6.2%)	0 (0%)	-
Hypertension	37 (32.7%)	19 (63.3%)	17 (53.1%)	1 (5%)	-
Chronic pulmonary disease (asthma, COPD)	4 (3.5%)	2 (6.7%)	2 (6.2%)	0 (0%)	-
Dyslipidemia	3 (2.7%)	1 (3.3%)	2 (6.2%)	0 (0%)	-
Diabetes mellitus	14 (12.4%)	7 (23.3%)	7 (21.9%)	0 (0%)	-
History of smoking	7 (6.2%)	4 (13.3%)	2 (6.2%)	1 (5%)	-
**COVID symptoms at presentation**					-
Cough	57 (50.4%)	21 (70%)	25 (78.1%)	11 (55%)	-
Shortness of breath	22 (19.5%)	8 (26.7%)	11 (34.4%)	3 (15%)	-
Dyspnea	33 (29.2%)	19 (63.3%)	13 (40.6%)	1 (5%)	-
Fever	48 (42.5%)	19 (63.3%)	19 (59.4%)	10 (50%)	-
Myalgia	33 (29.2%)	12 (40%)	15 (46.9%)	6 (30%)	-
Odinophagy	23 (20.4%)	11 (36.7%)	4 (12.5%)	8 (40%)	-
Rhinorrhea	14 (12.4%)	2 (6.7%)	6 (18.8%)	6 (30%)	-
Diarrhea	12 (10.6%)	7 (23.3%)	3 (9.4%)	2 (10%)	-
Vomit	3 (2.7%)	2 (6.7%)	1 (3.1%)	-	-
Ageusia	10 (8.8%)	5 (16.7%)	4 (12.5%)	1 (5%)	-
Anosmia	11 (9.7%)	5 (16.7%)	5 (15.6%)	1 (5%)	-
Asthenia	35 (31%)	16 (53.3%)	15 (46.9%)	4 (20%)	-
**Vital signs and laboratory measures**					-
Oxygen saturation > 95%	67 (59.3%)	1 (3.3%)	15 (46.9%)	-	-
D-dimer, mean (SD)	3.92 (19.89)	1.98 (5.44)	0.75 (0.47)	36.49 (72.34)	-
Creatinine, mean (SD)	0.94 (0.35)	1.04 (0.48)	0.87 (0.2)	0.87 (0.15)	-
Urea, mean (SD)	36.8 (17.15)	43.55 (22.55)	32.43 (10.01)	30.11 (6.85)	-
C-reactive protein, mean (SD)	63.7 (88.1)	142.67 (99.14)	75.72 (77.87)	6.02 (4.21)	2.77 (3.87)
**Chest CT category (Ground Glass Opacities)**					-
0 –no GG opacities	2 (1.8%)	0 (0%)	0 (0%)	2 (15.4%)	-
I–up to 25%	32 (28.3%)	3 (10%)	18 (56.2%)	11 (84.6%)	-
II– 25%–50%	23 (20.4%)	12 (40%)	11 (34.4%)	-	-
III–> 50%	18 (15.9%)	15 (50%)	3 (9.4%)	-	-
**Metabolite and ratio measures, mean (SD)**					
IDO (Kyn/Trp)	0.07 (0.07)	0.12 (0.1)	0.06 (0.03)	0.03 (0.01)	-
(Cit/Orn)	0.24 (0.11)	0.21 (0.08)	0.21 (0.09)	0.35 (0.12)	-
[(Kyn/Trp)/(Cit/Orn)]	0.39 (0.37)	0.6 (0.47)	0.36 (0.25)	0.11 (0.08)	-
(IDO/lysoPC a C18:0)	0.0027 (0.0038)	0.0046 (0.0056)	0.0020 (0.0014)	0.00092 (0.00080)	-
(Glu/PC aa C34:3)	11 (9.28)	14.93 (10.36)	11.24 (8.54)	4.73 (4.27)	-
(Asp/PC aa C34:3)	0.75 (0.56)	0.92 (0.46)	0.81 (0.68)	0.39 (0.31)	-
(IDO/PC aa C34:3)	0.01 (0.01)	0.01 (0.01)	0.01 (0)	0 (0)	-
C5	0.38 (0.31)	0.5 (0.39)	0.36 (0.24)	0.24 (0.18)	-
**COVID-19-related Outcomes**					
Recovered/discharged	77 (68.1%)	25 (83.3%)	32 (100%)	20	-
Required oxygen mask	18 (15.9%)	15 (50%)	18 (15.9%)	-	-
OTI + Mechanical Ventilation	18 (15.9%)	18 (60%)	18 (15.9%)	-	-
Tracheostomy	7 (6.2%)	7 (23.3%)	7 (6.2%)	-	-
Death	5 (4.4%)	5 (16.7%)	5 (4.4%)	-	0 (0%)

Plasma samples were obtained on all patients accrued to the study but processing errors in sample cryopreservation resulted in the loss of 5 samples leaving 77/82 (94%) of the samples fully evaluable.

Table 2 provides the most discriminating lipid ratios identified from the initial set of 186 metabolites.

**Table 2:** The most discriminating lipid ratios obtained from the data set of 186 metabolites.

METABOLITE RATIOS	AUC	T-tests
[(lysoPC a C26:0/PC ae C42:0)/PC aa C40:3]	0.99	2.10e-109
[(lysoPC a C26:0/PC aa C40:3)/PC ae C42:0]	0.99	2.10e-109
(lysoPC a C26:0/PC ae C42:0)	0.99	8.59e-115
(lysoPC a C28:0/PC ae C42:0)	0.99	1.48e-124
(lysoPC a C26:0/PC aa C40:3)	0.99	3.07e-105
(lysoPC a C28:1/PC ae C42:0)	0.99	9.78e-108
(lysoPC a C26:0/PC aa C34:2)	0.99	5.01e-93
(lysoPC a C26:0/PC aa C42:6)	0.99	8.91e-93
(lysoPC a C26:0/PC aa C36:3)	0.99	2.46e-93
(lysoPC a C26:0/PC aa C36:4)	0.99	3.05e-86
(lysoPC a C26:0/PC ae C42:1)	0.99	6.76e-99
(lysoPC a C26:0/PC aa C40:2)	0.99	1.19e-92
(lysoPC a C26:0/PC aa C42:5)	0.98	7.55e-83
(lysoPC a C26:0/PC aa C36:2)	0.98	1.05e-92
(lysoPC a C28:1/PC aa C32:3)	0.98	1.52e-97

Ratios utilized in multivariate ROC curve analysis 100-fold Cross Validation = 0.99, Permutation Test Statistics = p < 3.52e-6.

**Fig 2:**
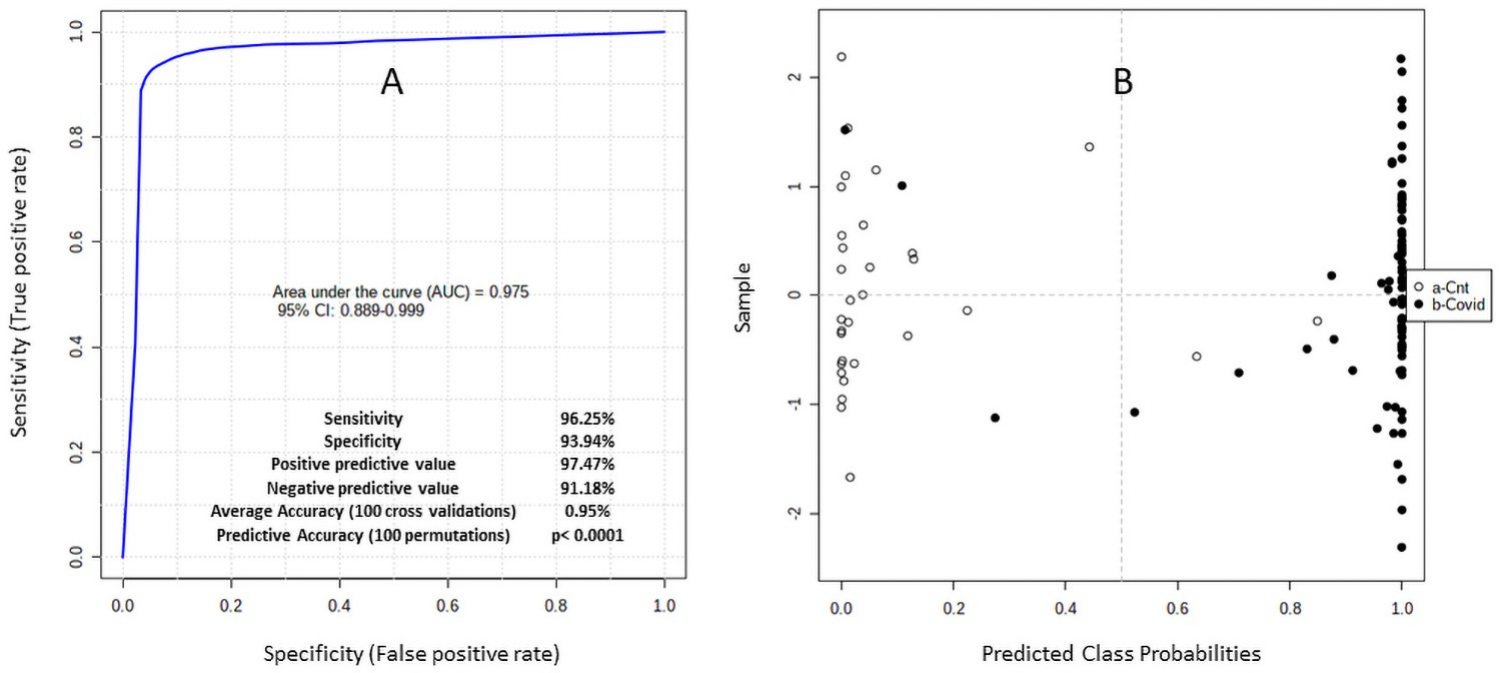
(A, B) reflect unsupervised clustering analysis using the most discriminating ratios that segregate controls (n = 31) from COVID-19 (+) patients (n = 77). The average accuracy based on 100 cross validations is 0.95 with an ROC AUC = 0.975 (95% CI 0.889–0.999) and permutation test statistic: p<0.0001.

These profiles segregated Covid-19 (+) patients from controls. Fig 2A provides the results of an unsupervised clustering analysis using the ratios from Table 2 to segregate controls (n = 31) from COVID-19 (+) patients (n = 77). Fig 2B shows the results of a multivariate analysis providing a receiver operator curve (ROC) AUC = 0.975 (95% CI 0.889–0.999).

With the ROC curve AUC = 0.975 that clearly identified a metabolic signature for Covid 19 infection, we included additional metabolites that were identified in the 77 Covid-19 (+) patient cohort to compare the signatures of 18 patients with mild infection to 59 patients with moderate or severe infection as defined by WHO criteria [6].

**Fig 3:**
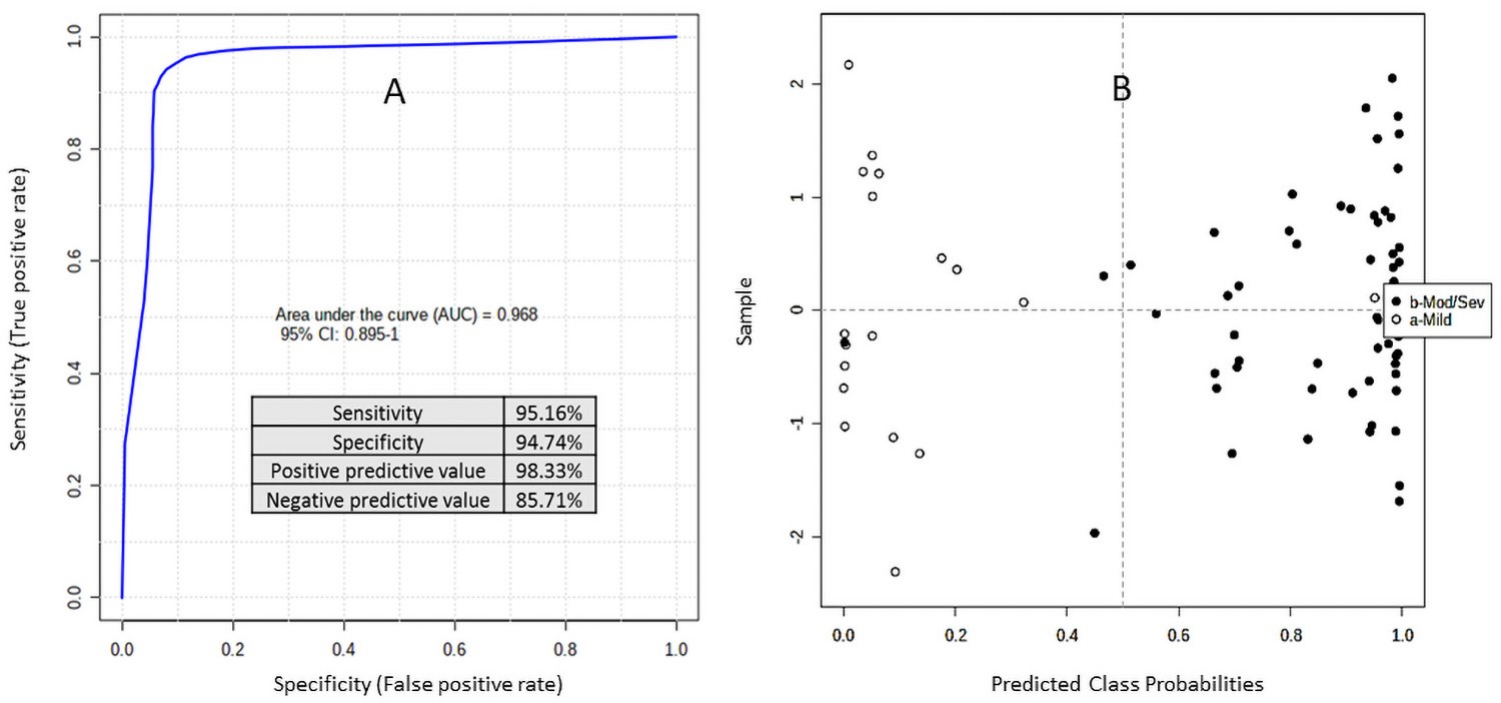
(A, B) provide base line predictions separating mild from moderate/severe using multivariate ROC Curve analysis applying the ratios obtained from the training set (Fig 2A and 2B): [(Glu/PC ae C42:1)/Taurine] and [IDO/(Cit/Orn)]/(PC ae C36:4). The average accuracy based on 100 cross validations is 0.90, Permutation Test (x500) statistics = p< 7.10e-05.

Fig 3A and 3B represents prediction, at base line, of patients with mild (n = 18) [empty circles] and moderate/severe Covid-19 (n = 59) [black circles]. This multivariate ROC Curve analysis used [(Glu/PC ae C42:1)/Taurine] and {[IDO/(Cit/Orn)]/(PC ae C36:4). The average accuracy based on 100 cross validations is 0.90, Permutation Test (x500) statistics = p< 7.10e-05. The ROC curve with an AUC = 0.968 (95% CI 0.895–1.0) sensitivity = 95.16%, Specificity = 94.74%, (+) predictive value = 98.33% and (-) predictive value = 85.71% indicates that plasma samples provide a robust predictor of Covid 19 morbidity and mortality.

**Fig 4:**
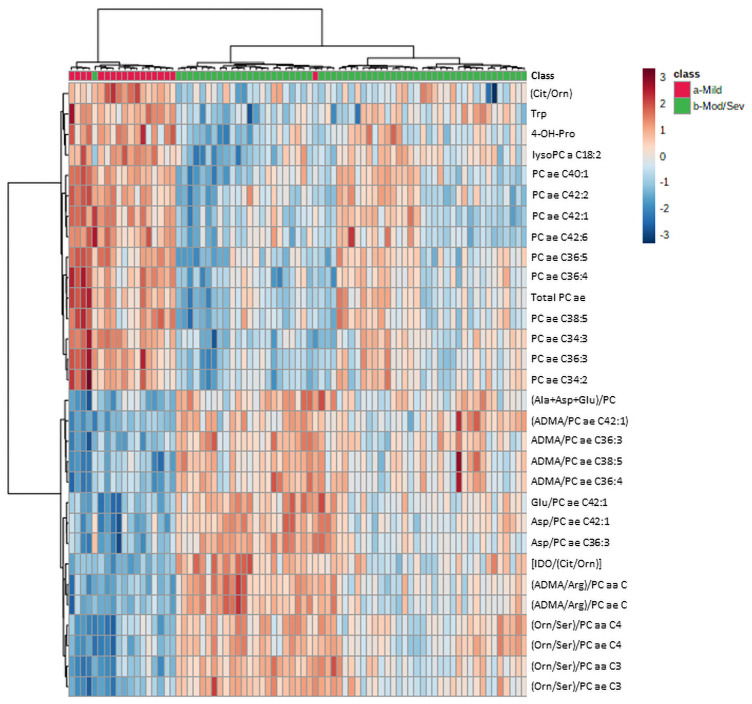
Heatmap of unsupervised clustering analysis using 30 most discriminating metabolites and ratios comparing mild (red) vs moderate/severe (green) Covid 19 outcomes.

Fig 4 provides an unsupervised clustering analysis as a heat map comparing mild (red) to moderate/severe (green) Covid infection. The results indicate that Covid 19 severity is associated with a decline in tryptophan (Trp) reflecting immune dysregulation. Early evidence that tryptophan metabolism regulated immunity (D) has more recently led to the observation that kynurenine/tryptophan ratios correlate with carbohydrate metabolism and cardio-metabolic risk [23] both associated with COVID-19 severity [24].

Alterations in liver function reflected by changes in the urea cycle (Cit/Orn), are consistent with the prior observations that patients with underlying liver disease are at significantly increased risk of morbidly and mortality from COVID-19 infection [25].

Increased inflammation associated with a decline in phosphatidyl cholines and a rise in lysophosphatidyl cholines, the result of phospholipase activation [26], reflects the inflammatory response to COVID-19 characteristic of hyper-immunity and an increased risk of morbidity and mortality as recently reported [27].

Finally, the results reveal increases in ADMA, a marker of epigenetic reprogramming that is associated with inflammation-related release of endothelial Nitric Oxide (NO) and has been shown to predict in-hospital mortality in COVID-19 patients [28].

While the measurement of individual metabolites provided insights into Covid-19 severity, ratios of analytes proved superior for the prediction of disease severity as they combined a multitude of metabolic perturbations into highly discriminating signatures.

**Fig 5:**
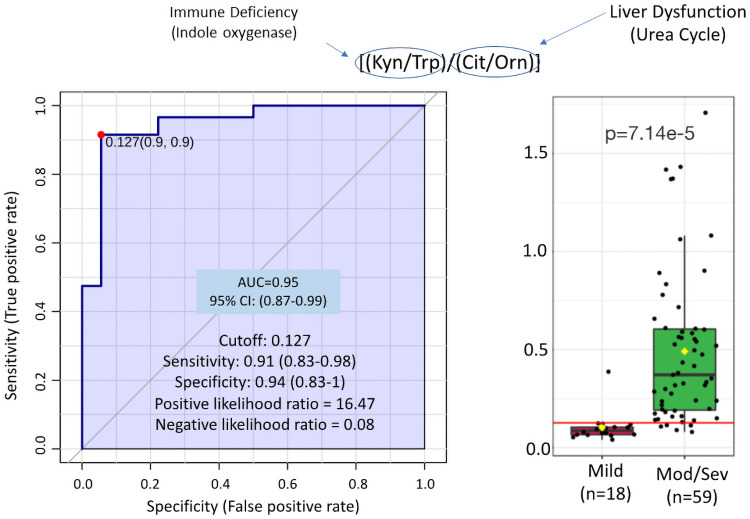
Ratio of immune dysfunction reflected by indole oxygenase activity (Kyn/Trp) over liver dysfunction reflected by ornithine transcarbamylase (Cit/Orn) discriminates patients with mild vs moderate/severe outcomes. 100-fold Cross Validation = 0.82, Predictive Accuracy (100 permutations) p = 1.00E-03.

Fig 5 provides the ratio of Kynurenine/Tryptophan (Kyn/Trp) divided by Citrulline/Ornithine (Cit/Orn) comparing mild (n = 18) to moderate/severe (n = 59) Covid-19 infection. By combining the IDO/TDO (indoleamine-2,3-dioxygenase (IDO) and tryptophan-2,3-dioxygenase (TDO) immune-ratio of Kynurenine/Tryptophan [29] with the liver-dysfunction-urea-cycle ratio of Citrulline/Ornithine [30] the receiver operator curve (ROC) AUC = 0.95 (95% CI 0.87–0.99) provides a more discriminating measure of disease severity confirming the multi-factorial nature of effective response to Covid-19.

Multivariate regression models were fit for each of four outcomes (moderate/severe vs. mild COVID-19); need for ventilator; complications besides pneumonia; and death), with independent variables including the metabolite measured, controlled for age, sex, and BMI. Models could not be run for several of the outcomes due to low numbers. However, our findings in Table 3 indicate that the ratio of glutamate and PC ae C34:3 was significantly positively associated with risk of developing moderate/severe COVID (OR = 1.283, 95% CI = 1.07, 1.68). The ratio of (Kynurenine/Tryptophan)/(Citrulline/Ornithine) (Kyn/Trp)/(Cit/Orn) was associated with increased risk of complications other than pneumonia (OR = 73.9, 95% CI 8.2, 1282.7) and need for ventilator (OR = 20.6, 95% CI = 3.1, 206.9). Valeryl-carnitine (C5) levels were strongly associated with risk for each outcome, with ORs ranging between 8.3 to 48.4. No other metabolite measures were good predictors for any of the other outcomes.

**Table 3:** Logistic regression models for selected COVID-19-related outcomes adjusted for age, sex, and BMI.

	Moderate/Severe COVID	Needed ventilator	Any complications besides pneumonia	Death
**Value**	**OR (95% CI)**	**OR (95% CI)**	**OR (95% CI)**	**OR (95% CI)**
**IDO (Kyn/Trp)**	**-**	**-**	**-**	**-**
**(Cit/Orn)**	**-**	**0.02 (0.00, 9.53)**	**0.01 (0.00, 5.10)**	**-**
**[(Kyn/Trp)/(Cit/Orn)]**	**-**	**20.62 (3.07, 206.96)** *	**73.91 (8.20, 1282.65)** *	**10.10 (0.51, 504.43)**
**(IDO/lysoPC a C18:0)**	**-**	**-**	**-**	**-**
**(Glu/PC ae C34:3)**	**1.283 (1.07, 1.68)** *	**1.05 (0.99, 1.12)**	**1.02 (0.95, 1.09)**	**1.04 (0.90, 1.12)**
**(Asp/PC aa C34:3)**	**28.61 (2.95, 891.34)** *	**2.03 (0.60, 5.93)**	**1.46 (0.34, 4.70)**	**1.964 (0.08, 41.91)**
**(IDO/PC aa C34:3)**	**-**	**-**	**-**	**-**
**C5**	**48.44 (1.62, 6094.69)** *	**8.35 (1.12, 71.91)** *	**21.51 (2.66, 248.73)** *	**8.95 (0.12, 1613.24)**

Note

* p <0.05.

As host response to viral infection reflects immune competence, we compared our Coronavirus COVID-19 signatures with those associated with the lentivirus HIV. It has been shown that certain subpopulations of HIV (+) individuals can tolerate the infection without progressing to AIDS. These individuals, known as “elites” [31] have been shown to have distinct metabolic features [5].

**Fig 6:**
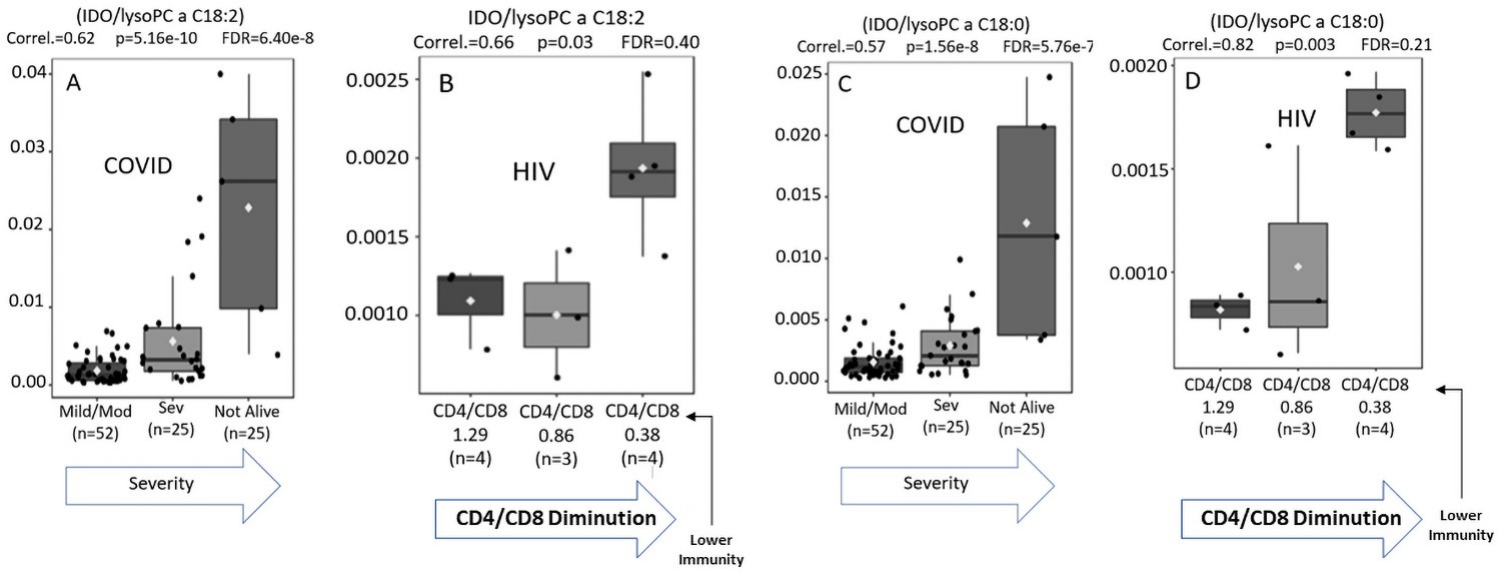
(A-D). Comparison of immune signatures for Covid 19 vs. HIV using immune IDO (Kyn/Trp) ratio divided by the inflammatory markers (lyso PC a 18:2 and 18:0) correlates Covid 19 severity with HIV progression.

Fig 6A–6D compare the metabolic signatures of patients of mild versus moderate/severe Covid-19 infection with those obtained from individuals with HIV. As the ratio of CD4/CD8 is an established parameter of HIV severity [22] we used cut offs of CD4/CD8 ratios to compare HIV severity with COVID-19 severity (mild/moderate vs. severe) using WHO criteria [6].

Using the immune (IDO) ratio divided by the lipid specie LysoPCa18:2, a measure of inflammation, we found a strong correlation between these two related but distinct retroviral infections.

**Fig 7:**
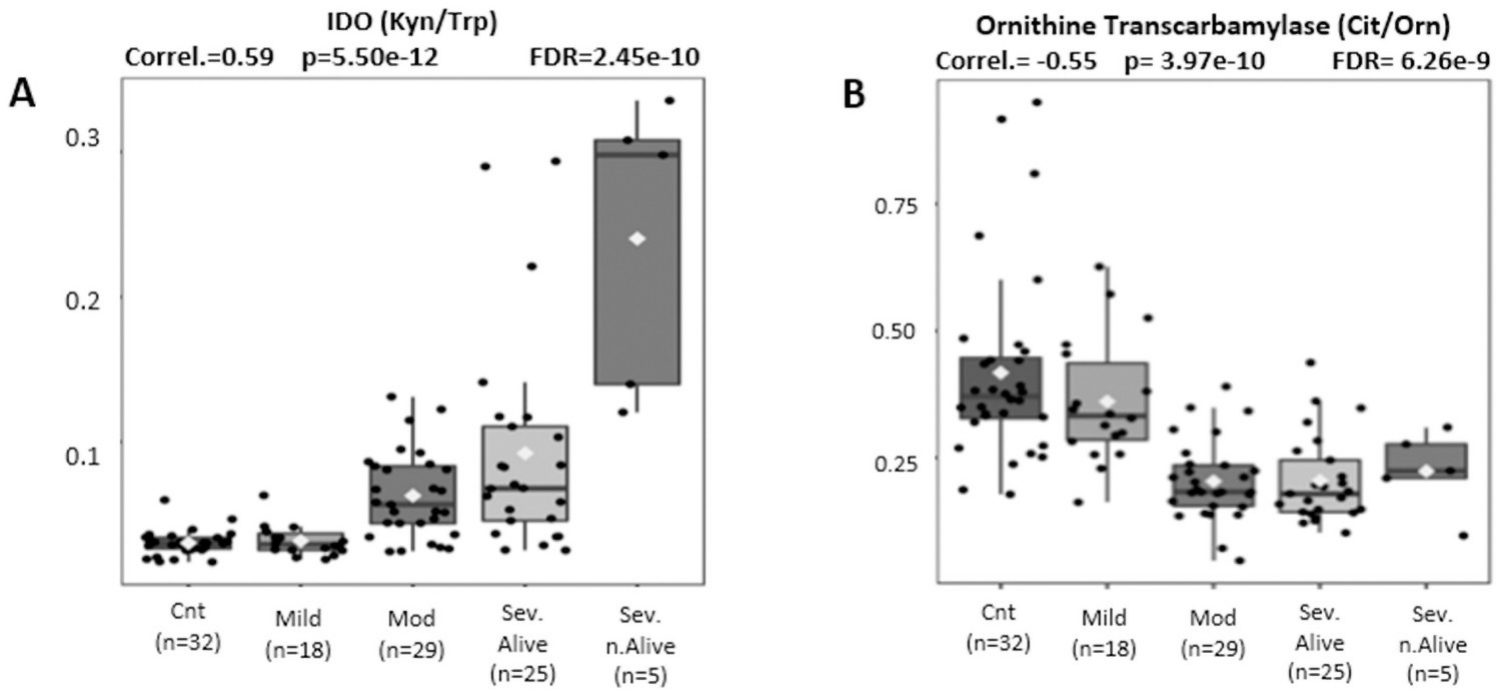
(A, B). Pearson Moment correlations of IDO activity (Kyn/Trp) and Ornithine transcarbamylase activity (Cit/Orn) for disease severity comparing controls, mild, moderate, and severe Covid patients.

Fig 7A and 7B correlate immune dysfunction measured by IDO (kyn/Trp) and liver dysfunction measured by ornithine transcarbamylase activity (Cit/Orn) with disease severity from controls to mild, moderate, severe, and lethal clinical outcomes.

**Fig 8:**
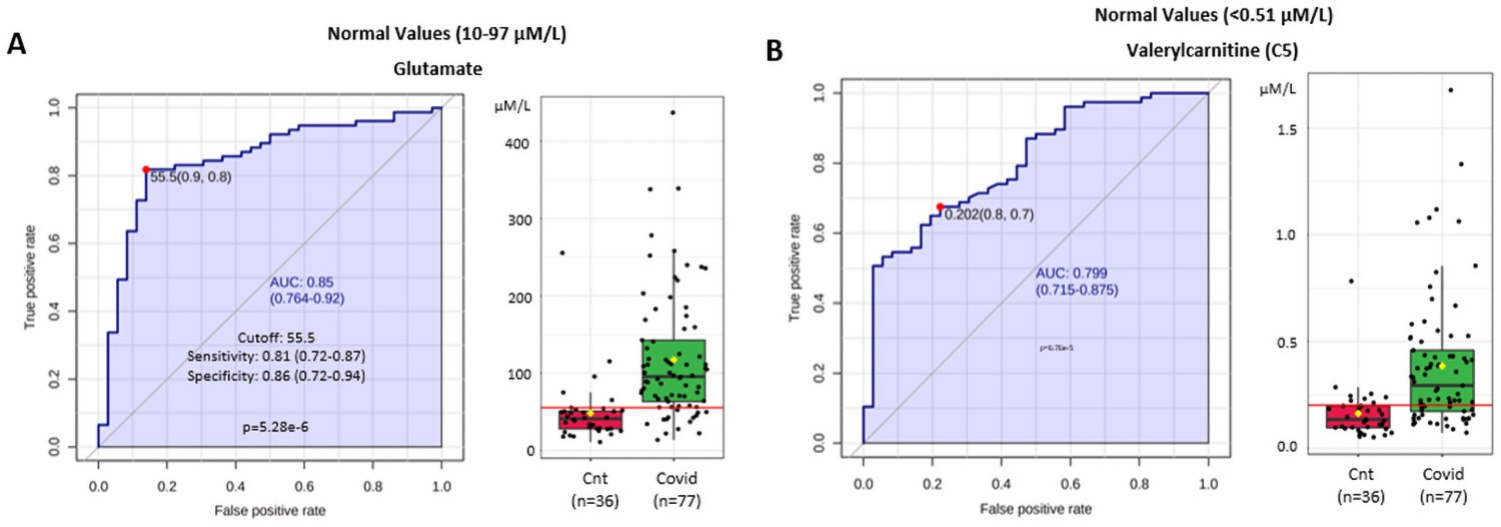
(A, B). Glutamate and Valeryl-carnitine (C5) concentrations comparing controls (red) n = 36 to Covid 19 patients (green) n = 77 provide ROC AUC = 0.85 (95% CI 0.764–0.92) and AUC = 0.799 (95% CI 0.715–0.875) respectively.

Fig 8A and 8B compare metabolic signatures for controls vs Covid 19 (+) patients using measures of glutaminolysis (Glutamate) and mitochondrial dysfunction reflected by organic acidemia (Valeryl-carnitine C5) to provide ROC curves with an AUC = 0.85 (95% CI 0.764–0.92) and an AUC = 0.799 (95% CI 0.715–0.875) respectively that clearly distinguish the two groups.

Our findings are in agreement with the recent study reported by Herrera-Van Oostdam et al. [27] that identified immune-metabolic signatures as predictors of COVID-19 progression to sepsis. Among the similarities are perturbations in the Kynurenine/Tryptophan ratios, changes in phosphatidylcholine / lyso-phosphatidylcholine ratios and alterations in valeryl-carnitine. Italian investigators using targeted lipidomics have shown that COVID-19 is associated with alterations in sphingolipids, specifically ceramides [28].

The association between COVID-19 severity and obesity, diabetes, and cardiovascular disease [4] suggests that metabolic stress contributes to the morbidity and mortality of this infection [32, 33]. Recognizing that effective immune response draws upon numerous physiologic reserves, we found that COVID-19 severity could be predicted using algorithms that incorporate multiple aspects of altered metabolism. Combining lipid ratios with measures of liver dysfunction (Citrulline/Ornithine); mitochondrial dysfunction (Valerylcarnitine), glutaminolysis and immune response (Kyn/Trp) provided the most discriminating signatures.

To examine whether these findings extended to other infections, we compared the COVID-19 signatures with those associated with HIV infection. Correlations between the severity of HIV measured as CD4/CD8 ratios with the severity of COVID-19 by WHO criteria suggest that defense against these two distinct but related retroviral infections reflect shared features of human immune response.

Our findings suggest that host factors play an important role in COVID-19 pathogenicity. Metabolic changes may predispose certain individuals to higher risk of morbidity and mortality. In keeping with the recent findings of other investigators in the field, metabolomic analyses may provide important tools as we confront new challenges in the ongoing COVID-19 pandemic.

### Limitations of the study

The study was undertaken as an exploratory analysis with patients accrued from a single institution in southwestern Brazil during the COVID-19 resurgence (second wave). Newly diagnosed patients were compared with suffering more severe illness. We recognize that pharmacologic interventions in the severe group including dexamethasone, supplemental oxygen, heparin, antibiotics and two patients who received tocilizumab could have had an impact on the observed metabolic signatures. No patients received Remdesivir. Future studies will accrue patients at first presentation to control for these variables.

Our control group consisted of PCR negative, healthy hospital staff who were regularly screened as part of hospital policy. Our controls could also have included patients presenting with respiratory symptoms who were then proven PCR negative, and this will be examined in future studies.

The principal limitation of the study was sample size that precluded a more thorough examination of clinical parameters of severity against biochemical measures. Logistic regression did reveal correlations, but the confidence intervals were large leaving many of the findings as hypothesis-generating.

## Conclusions

We conclude that the severity of COVID-19 infection represents the complex interaction between the organisms’ innate pathogenicity and the hosts’ response. Commonalities between COVID-19 and HIV suggest a critical role for the host’s metabolic wellbeing as a determinant of clinical severity in these and perhaps many infectious processes. The metabolic signatures associated with COVID-19 severity may offer new diagnostic and prognostic determinations that could lead to novel interventions for the treatment or prevention of the biochemical frailties that predispose individuals to severe disease.
